# Validation of Azure Kinect for Upper Limb Motion Analysis Under Optimal and Suboptimal Conditions

**DOI:** 10.3390/s26134098

**Published:** 2026-06-27

**Authors:** Gabriele Fassina, Serena Cerfoglio, Michele Rigucci, Alessandra Pedrocchi, Veronica Cimolin, Emilia Ambrosini

**Affiliations:** 1 NearLab, Department of Electronics, Information and Bioengineering, Politecnico di Milano, 20133 Milan, Italy; gabriele.fassina@polimi.it (G.F.); michele.rigucci@mail.polimi.it (M.R.); alessandra.pedrocchi@polimi.it (A.P.); 2 Movlab, Department of Electronics, Information and Bioengineering, Politecnico di Milano, 20133 Milan, Italy; serena.cerfoglio@polimi.it (S.C.); veronica.cimolin@polimi.it (V.C.); 3 IRCCS Istituto Auxologico Italiano, San Giuseppe Hospital, Piancavallo, 28823 Verbania, Italy

**Keywords:** markerless motion capture, Azure Kinect, kinematics, upper limb, validity study

## Abstract

Assessment of upper-limb kinematics is essential in clinical practice for diagnosis, rehabilitation monitoring, and treatment personalization. Markerless Motion Capture (MMC) systems, such as the Microsoft Azure Kinect (AK), offer a low-cost and time-efficient alternative to marker-based systems. However, while AK accuracy has been extensively studied for lower-limb movements, its performance for upper-limb analysis—especially under clinically relevant, suboptimal conditions—remains underexplored. This study aims to validate AK for upper-limb motion tracking against a gold-standard optoelectronic system under optimal and suboptimal conditions. Sixteen healthy adults performed ten upper body motor tasks in three scenarios: optimal setup, seated posture with table occlusion, and increased camera distance. Joint angles were compared using normalized Root Mean Squared Error (nRMSE) and Pearson’s correlation coefficient. Performance Indicators (PIs) including Range of Motion (ROM), smoothness, and Time to Peak Velocity (TTPV) were also evaluated. AK accurately captured movements performed within the camera plane, with median nRMSE below 20% in optimal conditions and no significant degradation in suboptimal setups. In contrast, movements occurring on planes perpendicular to the camera were poorly captured. ROM estimation was acceptable and highly reproducible, while TTPV showed moderate-to-poor reliability and smoothness deviated substantially from the reference system. These findings suggest that careful attention to Kinect positioning is essential to ensure effective acquisitions, even in suboptimal scenarios. Future research should evaluate AK validity in clinical populations and explore the effects of system interference in multi-device setups.

## 1. Introduction

The study of human movement is crucial across various domains, including entertainment, athletic performance assessment [1], ergonomics and worker safety [2,3], and clinical applications. In the medical field, accurate and reliable measurement of human motion is essential to assess posture and balance control, diagnose orthopedic and neurological conditions, and improve rehabilitation treatments [4]. While traditional motor assessments rely on qualitative clinical scales, more refined evaluations emphasize the need for more quantitative parameters (e.g., joint kinematics), driving the development of technologies capable of capturing such data [5].

Among the latter, marker-based optoelectronic systems (OSs) are widely regarded as the gold standard [6], achieving millimeter-level accuracy in tracking 3D marker positions [7]. However, despite their precision, they suffer from notable drawbacks, including high costs, the need for dedicated laboratory set-up and specialized personnel [8]. In addition, marker placement and camera calibration processes extend the duration of the test [6]. Finally, acquisition conditions may induce the Hawthorne effect, in which subjects alter their natural movement patterns due to the awareness of being observed [9], hindering ecological assessment [10].

To overcome these limitations, research has increasingly focused on markerless motion capture approaches. These systems are generally more portable and require substantially simpler setup procedures, enabling faster acquisition workflows and reducing the need for specialized operators. Furthermore, the possibility of using unobtrusive camera configurations may help mitigate the Hawthorne effect, thereby promoting more ecological movement assessment. Recently, multiple RGB cameras enhanced with state-of-the-art artificial intelligence (AI) tools are gaining relevance [11,12]. In this direction, the most prominent solution is OpenCap [13], a platform to perform 3D Markerless Motion Capture (MMC) by using multiple cameras with non-parallel optical axes. Although such approaches demonstrate promising results, they require multiple cameras and calibration procedures and cannot provide real time feedback, which makes RGB-D sensors the predominant tool for performing 3D MMC [4]. These sensors are equipped with depth retrieval capabilities based on Time-of-Flight (TOF) or the structured light principles [3], enabling the reconstruction of 3D coordinates of anatomical landmarks, referred to as keypoints.

Among other devices, Kinect^®^ cameras are the most widely used sensors for MMC [11]. They were initially designed for entertainment purposes but later adopted by the scientific community for diverse motion capture applications [14]. After Kinect V1 and V2, which were discontinued in 2019, the Azure Kinect (AK) camera was released. This combines TOF technology with a custom-developed convolutional neural network (CNN) for body tracking [7].

Kinect sensors have been employed to assess various medical conditions, particularly those diagnosed through characteristic motor signs. Parkinson’s Disease (PD) is the condition where MMC has been most extensively utilized, primarily due to its ability to effectively measure gait spatio-temporal parameters [15,16]. Similarly, hemiplegic conditions can also benefit from MMC [17]. MMC also proves valuable in cases where motor signs are not the core symptoms for diagnosis, such as Autism Spectrum Disorder (ASD) [18]. Within the medical domain, markerless motion capture technologies are also gaining attention for developing AI-powered classifiers to distinguish between healthy and pathological motions [8] and for action recognition tasks [19]. The above-mentioned clinical applications pose considerable challenges for effective motion capture. For instance, post-stroke assessments often require pose tracking in the sitting condition, such as during the administration of clinical scales like the Fugl-Meyer Assessment (FMA) scale and the Wolf Motor Function Test (WMFT) [20]. It is therefore crucial that the sensor can reliably track human pose even when subjects are seated. This requirement is further emphasized in wheelchair-sitting subjects [21]. Moreover, mainly in pediatric applications, acquisitions often occur in unconstrained environments where children can move freely to evaluate their tendency to explore the space [22]. In such scenarios, MMC systems must ensure consistent tracking, even when subjects do not conform to manufacturer-recommended positions.

Given the stringent accuracy requirements of medical applications, validating motion tracking technologies is crucial. Most existing studies have focused on lower-limb tracking, primarily to assess the effectiveness of Azure Kinect in walking tasks. Studies such as [1,7], and [23] evaluated the device’s ability to estimate joint positions, concluding that AK outperforms its predecessor, particularly for lower extremity joints. However, higher errors were observed for occluded joints and during sudden movements. Yeung et al. [24] validated AK by comparing lower-limb joint angles with those obtained from an optoelectronic system during gait analysis. They reported improved accuracy when the camera was positioned laterally to the walking direction. However, sufficient accuracy was also observed when the subject walked towards the camera. Other studies [25,26] examined gait spatio-temporal parameters, such as step and stride lengths and times, and confirmed AK’s accuracy, especially when the camera was positioned diagonally to the walking direction, consistently with [24]. Furthermore, Thomas and colleagues [27] investigated AK’s performance in tracking the Sit-to-Stand Test (STS), finding high waveform similarity (measured via Pearson’s correlation coefficient) but also large Root Mean Square Errors (RMSEs), indicating inaccuracies in certain phases of the task. Finally, Jo et al. [28] extended the evaluation to full-body functional movements, including squats, lunges, and reaching tasks, reporting good agreement for hip and knee angles but limited accuracy for ankle angles and occluded segments.

While most validation studies have concentrated on lower-limb motion analysis, a smaller number have investigated the accuracy of markerless motion capture systems for upper-limb tracking. Before the release of Azure Kinect, some studies validated the performance of Kinect V2 for upper-limb movements. Specifically, Cai et al. [29] evaluated Kinect V2 during functional upper-limb tasks and reported that movements performed in the sagittal plane, i.e., perpendicular to the camera plane, exhibited the largest errors (approximately 10°), whereas shoulder abduction and adduction angles showed lower errors (approximately 6°). These findings highlighted the strong dependence of tracking accuracy on the specific motor task being performed. In a subsequent study, Cai et al. [30] further demonstrated that measurement error was unaffected by the subject-to-camera distance, whereas camera placement had a substantial impact on tracking accuracy. Similarly, Scano et al. [31] validated Kinect V2 during the execution of upper-limb motor primitives and reported comparable results, with larger errors observed for elbow flexion than for shoulder kinematics. Finally, Faity et al. [32] evaluated Kinect V2 under conditions designed to mimic post-stroke movement patterns by applying an external load to the hand of healthy participants. Their results showed relative errors of approximately 20% for joint-angle estimation, indicating moderate agreement with the reference system. Based on these findings, the authors concluded that Kinect V2 may be more suitable for global motion assessment than for accurate joint-level kinematic analysis.

Following the introduction of Azure Kinect, a limited number of studies investigated whether the new sensor and its deep learning-based body-tracking framework improved upper-limb motion tracking performance. Ozsoy et al. [33] demonstrated that Azure Kinect provides greater repeatability and accuracy than Kinect V2 in the estimation of shoulder angles. Likewise, Ivorra et al. [34] confirmed the ability of Azure Kinect to reconstruct shoulder abduction accurately but highlighted persistent difficulties in estimating shoulder rotation, likely due to keypoint occlusions. Intermediate results were observed for shoulder flexion, whereas elbow flexion exhibited the largest errors, particularly when the movement occurred outside the camera plane. Lastly, Brambilla et al. [6] conducted a comprehensive study on various reaching tasks, corroborating Ivorra’s findings that shoulder and elbow flexion angles exhibited the highest errors. Additionally, they reported that partial occlusion of the legs by a table significantly reduced accuracy. However, table occlusion was not investigated under seated conditions, despite this representing a common setup in clinical assessments. Furthermore, the authors analyzed the effect of camera placement and found that the best results were obtained when movements occurred within the camera plane. Nevertheless, while camera orientation was varied, the subject-to-sensor distance remained fixed throughout the experiments. This differs from several clinical and rehabilitation scenarios, where patients are free to move within the environment and cameras must reliably capture motion across varying distances. Therefore, the impact of seated table occlusion and subject-to-camera distance on motion capture accuracy remains insufficiently explored.

In conclusion, despite the frequent occurrence of suboptimal tracking conditions in clinical practice, there is a lack of research validating the accuracy of the AK under such conditions. Indeed, all related studies are conducted with standing subject at the recommended distance from the camera. This work aims to address this gap by conducting a validity study of the AK’s performance for upper-limb motion tracking with respect to a gold-standard optoelectronic system (OS) in a group of healthy volunteers. The validation was assessed both in optimal and suboptimal conditions commonly encountered in clinical settings, i.e., with individuals seated at a table and at increased camera distance. The results of this study should provide a foundation for sensorizing assessments commonly conducted in routine clinical practice. To reach this aim, the validation encompasses both the sensor and its body-tracking Software Development Kit (SDK), ensuring that non-technically trained personnel can use the device with a fully automated workflow.

## 2. Material and Methods

### 2.1. Participants

The study involved healthy young adults who participated on a voluntary basis. Inclusion criteria encompassed individuals aged 18 to 65 years, with no diagnosed musculoskeletal or neurological disorders. Subjects taller than 190 cm were excluded to ensure compatibility with the control volume of the OS. The expected sample size was estimated between 15 and 20 participants, based on the number of subjects commonly used in similar validation studies [24,25,26,27]. The study received approval from the Ethical Committee of Politecnico di Milano (approval number 32/2023) and was carried out in accordance with the principles of the Declaration of Helsinki. All participants provided informed consent at enrollment and were allowed to withdraw from the study at any time.

### 2.2. Experimental Setup

The experimental setup for this study consisted of an Azure Kinect (AK) camera, and a gold standard OS (BTS-400 DTX) with eight cameras. The AK was mounted on a tripod at a height of 1 m from ground, positioned frontally to the subject—as suggested in [6]—and aligned with the center of the optoelectronic system’s control volume.

#### 2.2.1. Azure Kinect

The Azure Kinect (Microsoft Inc., Redmond, WA, USA) is an RGB-D camera equipped with a Time-of-Flight (ToF) depth sensor that can operate in five distinct modes, each differing in acquisition volume, range, and resolution. In this study, the Binned Wide Field of View (WFOV-binned) option was selected, offering a 120° × 120° field of view—the widest available—as a large acquisition volume is essential to capture the full range of motion of upper-limb movements. Although this mode has lower resolution than the unbinned option, it allows acquisition at up to 30 Hz, compared to the 15 Hz limit of the unbinned modality. This higher frame rate facilitates capturing faster movements and tremors, which can be important in certain clinical applications.

**Figure 1:**
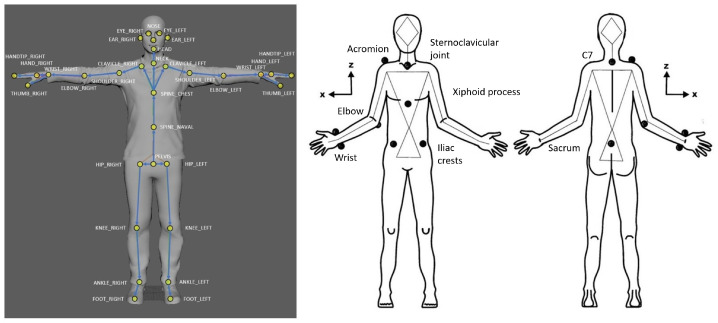
On the left, the 32 Azure Kinect keypoints are reported, as defined on the manufacturer website (https://azure.microsoft.com/it-it/products/kinect-dk, accessed on 23 July 2025), while on the right the marker placement for the optoelectronic system is outlined.

The Azure Kinect is endowed with its own body-tracking SDK, which employs deep neural networks (DNNs) and convolutional neural networks (CNNs) to determine the 3D positions of 32 keypoints [7], as illustrated in Figure 1, left panel. The body-tracking SDK version 1.4.1 was used for data acquisition, integrated into a Python-based graphical user interface (GUI) for managing data storage. Python 3.13.7 was used for this implementation. In this study, the Azure Kinect sensor’s performance was evaluated exclusively through the SDK, ensuring that it can be used in clinical practice without requiring additional post-processing to retrieve keypoints. The device was connected via a USB-3.0 port to a PC (Intel Core i7 CPU @2.60 GHz; 16 GB RAM; NVIDIA GeForce GTX 1660 Ti GPU). The sampling rate of the Azure Kinect is not constant, as it is affected by fluctuations resulting from the use of GPU-based body-tracking algorithms. In our setup, the acquisitions exhibited a sampling rate of 23±3 Hz.

#### 2.2.2. Optoelectronic System

An 8-camera optoelectronic motion capture system (BTS SMART DX 400, BTS Bioengineering, Milan, Italy) was adopted as the gold standard. This fully validated system is commonly employed in both clinical and research settings. Ten spherical retro-reflective markers were manually placed on specific anatomical landmarks to approximate the keypoints identified by the Azure Kinect body tracking SDK. Specifically, a reduced version of the marker set proposed by Menegoni et al. was adopted [35]. Two markers were placed on the wrist (styloid processes), one on the elbow (lateral epicondyle), one on the acromion, one on the sternoclavicular joint, and one on the xiphoid process. Additional markers were positioned on the iliac crests, the spinous process of the C7 vertebra, and the sacrum. The right panel of Figure 1 illustrates the adopted marker placement. This reduced marker set was selected to minimize the number of reflective markers required for joint-angle computation, thereby limiting potential interference between the infrared signals emitted by the optoelectronic system and the Azure Kinect sensor. Notably, no markers were placed on the hands, as the wrist was considered a more reliable anatomical reference for reconstructing the forearm segment. Furthermore, no analysis of end-effector kinematics was performed, since direct position comparisons were not considered reliable due to the imperfect spatial correspondence between optoelectronic markers and Azure Kinect keypoints. The acquisition frequency of the OS was set at 100 Hz. Before starting each subject’s acquisition, the system was calibrated to ensure optimal functioning and accuracy.

### 2.3. Experimental Protocol

This work presents a validation study in which participants were concurrently acquired by an Azure Kinect camera and the optoelectronic system, while performing upper body motor tasks. The experimental protocol was designed to include movements across different planes and involving the various degrees of freedom of the upper-limb joints. The choice of the tasks, the marker placement, and the processing pipeline were all defined in line with approaches commonly adopted in the field [36]. This design ensures reproducibility and provides a potential basis for establishing a standardized protocol for upper-limb kinematic assessment.

#### 2.3.1. Motor Tasks

**Figure 2:**
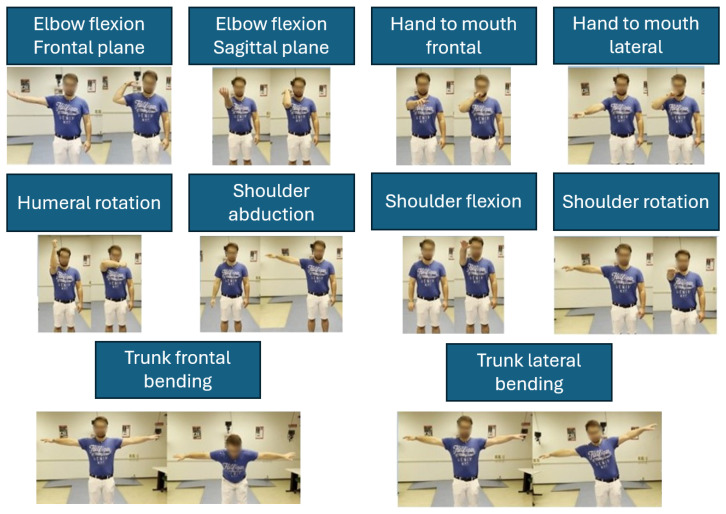
Upper-limb motor tasks included in the experimental protocol. Pictures are taken from the camera’s point of view.

The study encompassed ten motor tasks focusing on arm and trunk movements, illustrated in Figure 2. Specifically, upper-limb motions included shoulder abduction, shoulder flexion, shoulder rotation, elbow flexion (performed with the arm extended in both the frontal and sagittal planes), and humeral rotation. For trunk movements, frontal and lateral bending movements were analyzed. Additionally, two hand-to-mouth tasks, performed both frontally and at 60° with respect to the camera plane, were incorporated to validate the Azure Kinect body tracking system during a functional movement commonly evaluated in clinical assessments. Separate reaching tasks were not included, as they were considered to be encompassed within the hand-to-mouth movement pattern. All movements were performed using the participant’s dominant arm. No comparison between limbs was conducted, as no substantial differences in tracking performance between sides were expected in the healthy population considered in this study.

#### 2.3.2. Testing Conditions

**Figure 3:**
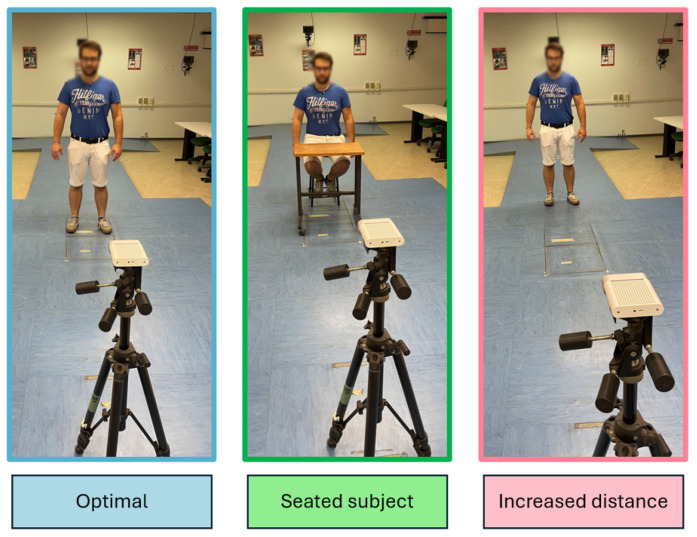
Testing conditions. The optimal condition, as indicated by the manufacturer, implies that the subject stands 2.5 m from the camera. In the seated subject condition, the person is sitting on a stool placed behind a table that is 2.5 m from the camera. In the increased distance condition, the subject is standing 3.5 m from the camera.

Participants performed all motor tasks described in Section 2.3.1 under three distinct experimental conditions: optimal, seated subject, and increased distance. In the optimal condition, participants stood 2.5 m from the Azure Kinect camera, facing it directly with no occlusions between them and the camera. In fact, this is the tracking condition suggested by the manufacturer. In the seated subject condition, participants were seated on a stool at a table, which introduced an obstacle between the subject and the camera. The stool was positioned such that its center was at a distance of 2.5 m from the camera. This condition was designed to evaluate the Azure Kinect body tracking system’s ability to accurately capture movements despite the subject being seated and the presence of potential occlusions. Finally, in the increased distance condition, participants stood at a distance of 3.5 m from the camera, exceeding the nominal range specified by the manufacturer. This condition aimed to examine the tracking system’s capability to capture movements when the subject was located at a greater distance. Figure 3 reports the three testing conditions.

#### 2.3.3. Acquisition Protocol

Subjects repeated each motor task six times in each condition at a self-selected speed. Figure 3 illustrates the subject’s posture at the beginning of the trial. In particular, subjects were asked to adopt the N-pose in cases when they were standing and to sit with arms lying at their side in the seated subject condition. To synchronize the motion data obtained from both acquisition systems, subjects performed a predefined gesture before the first repetition of each task. Specifically, starting from the initial pose, they lifted their right arm to 90° on the frontal plane and maintained this position for 2 s.

### 2.4. Data Analysis

#### 2.4.1. Joint Angles Calculation

**Figure 4:**
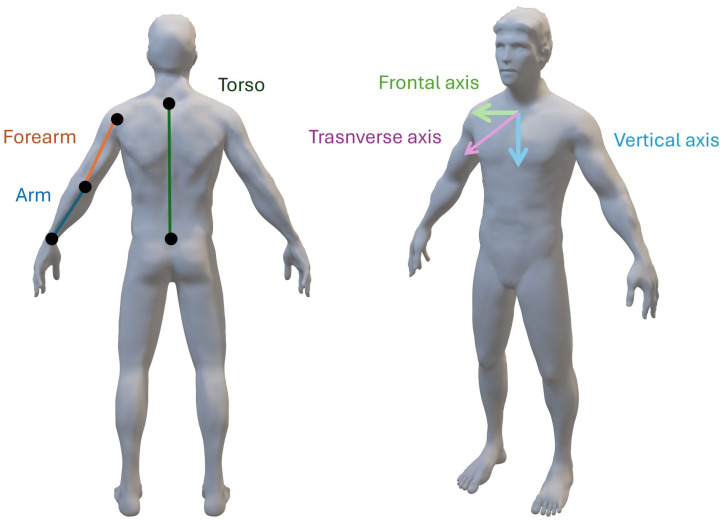
The left panel illustrates the body segments used for upper-limb kinematic analysis, namely torso, arm, and forearm, together with the associated anatomical landmarks. The right panel depicts the anatomical reference system, including the frontal, transverse, and vertical axes adopted for movement description.

Both the OS and AK return 3D coordinates of the anatomical landmarks. To avoid introducing differences in the estimated kinematic profiles that could arise from system-specific filtering rather than from the measurement systems themselves [32], motion data from both sensors were analyzed using the same pipeline, which was implemented in MATLAB, R2024B (Mathworks Inc., Natick, MA, USA). First, a cubic polynomial interpolation was applied to the optoelectronic data to compensate for missing values. This procedure was not necessary for AK data, as its embedded deep learning algorithm estimates the position of all keypoints for every acquired frame. Next, 3D vectors representing anatomical segments were derived from the keypoints coordinates. Specifically, the forearm was defined as the vector connecting the elbow to the wrist, with the midpoint between the two placed markers chosen to identify the wrist joint for the optoelectronic system; the arm was represented as the vector from the shoulder to the elbow; the trunk was defined as a vector passing through the spine, i.e., connecting the Sacrum with the C7 vertebra for the OS, and the pelvis with neck keypoints in the AK frame. Additionally, an anatomical reference frame was defined to permit joint-angle computations: a frontal axis was established by connecting the shoulder marker with the sterno-clavicular one in the OS system and the right clavicle keypoint with the neck in the AK body tracking system; a vertical axis was identified by connecting the xiphoid process to the sterno-clavicular joint for the optoelectronic system and the spine naval keypoint with the spine chest one in the AK body tracking framework during the initial N-pose; finally, the transverse axis was defined to be orthogonal to the previous ones. Figure 4 represents the vectors used to define both anatomical segments and the anatomical reference frame.

For each motor task, the angles with an expected range of motion higher than 20° were analyzed. Joint angles were defined following the International Society of Biomechanics (ISB) convention [37] and computed according to Equation (1), where u and v represent the two vectors forming the angle of interest. Given the marker minimization principle adopted to reduce cross-talk effects, different marker selections were used to define body segments in the two acquisition systems. Although this choice may introduce systematic measurement offsets between AK and OS, it was considered preferable to the use of redundant markers, which could increase soft-tissue artifacts and cross-talk, potentially compromising the reliability of the estimated joint kinematics. Moreover, several keypoints are estimated by the SDK through an internal biomechanical model and may be located away from palpable bony prominences or even represent internal anatomical locations. Therefore, an exact spatial correspondence between Azure Kinect keypoints and optoelectronic markers cannot be established, and the selected marker set should be interpreted as the closest anatomical approximation available. Furthermore, this methodological difference is expected to have a more limited impact on joint-angle computation than on the estimation of absolute joint positions, which is therefore not performed in this work. Table 1 details the specific angles analyzed for each motor task and the corresponding vectors used for their computation.

(1) $$\theta =arccos\left(\frac{u\cdot v}{\left||u||\cdot ||v|\right|}\right)$$

A 4th-order low-pass Butterworth filter with a cutoff frequency of 4 Hz was applied to reduce high-frequency noise. We chose a cut-off frequency of 4 Hz because it preserves the dominant frequency content of the investigated upper-limb movements while reducing high-frequency measurement noise. Preliminary spectral analyses indicated that frequencies above 4 Hz contributed minimally to the overall signal energy. To enable point-wise comparison, the computed angles were resampled so that both data streams contained the same number of samples. Since the optoelectronic system recorded data at 100 Hz, while the Azure Kinect system operated at 23 ± 3 Hz, both datasets were downsampled to 20 Hz. The signals from both acquisition systems were then synchronized using the predefined synchronization gesture performed at the beginning of each trial. The synchronization procedure relied on the temporal alignment of characteristic kinematic events within the shoulder elevation trajectories. In particular, the first peak of the synchronization gesture was identified in both signals and used as a reference event for temporal alignment. Since this event corresponds to a change in movement direction, the synchronization relies on a distinct kinematic feature of the movement rather than on the overall similarity of the angle trajectories, making it less sensitive to differences in waveforms between systems. After synchronization, the angle data were segmented to isolate the six task repetitions. Transition moments between consecutive repetitions were identified by detecting stationary points in the low-pass-filtered angle signals measured by the OS, i.e., points at null angular velocity. For each task, the angle with the widest ROM measured through the OS was used to separate the repetitions. Finally, to perform comparison across repetitions, time normalization was performed, expressing motion execution as a percentage of movement duration.

The analysis was also extended to joint angular velocity profiles, obtained as the first derivative of the joint-angle trajectories. Because movement duration was not considered after time normalization, the derivatives represent normalized angular velocity profiles rather than absolute angular velocities (°/s). Nevertheless, these profiles provide information on the temporal evolution of joint kinematics and allow the same waveform-based comparisons performed on the joint-angle trajectories, detailed in the following paragraphs.

#### 2.4.2. Accuracy Metrics

The Root Mean Squared Error (RMSE) and the normalized Root Mean Squared Error on the joint angles was used to quantify the discrepancy between AK and OS kinematic profiles, as detailed, respectively, in Equations (2) and (3), where *n* refers to the number of samples in the current repetition after time normalization, and θ refers to the angle acquired by the OS and AK, respectively. Specifically, the RMSE was normalized on the Range of Motion (ROM) computed on the OS data in the current task repetition.

(2) $$RMSE=\sqrt{\frac{1}{n}\sum_{i=1}^{n}{\left(\theta_{OS}-\theta_{AK}\right)}^{2}}$$

(3) $$nRMSE=\frac{1}{ROM_{OS}}\sqrt{\frac{1}{n}\sum_{i=1}^{n}{\left(\theta_{OS}-\theta_{AK}\right)}^{2}}\cdot 100$$

The Pearson’s correlation coefficient was subsequently computed in order to assess the strength and direction of linear relationships between the angles acquired by the OS and AK. The MATLAB corrcoef function was employed to derive this outcome metric.

Both nRMSE and Pearson’s correlation coefficient were computed for each repetition, and the median and interquartile range across subjects are reported. The combined use of these two metrics enables a more comprehensive assessment of waveform agreement: Pearson’s correlation coefficient quantifies the similarity in waveform shape, whereas RMSE and nRMSE capture discrepancies and potential systematic biases that may not be detectable through correlation analysis alone.

The same analysis was repeated for the joint angular velocity profiles, for which RMSE, nRMSE, and correlation coefficients were computed. This additional analysis was performed to reduce the influence of potential constant offsets arising from marker placement or anatomical calibration errors. Because differentiation is insensitive to constant angular offsets, velocity profiles provide a complementary assessment of the agreement between systems, focusing on the temporal evolution of the movement rather than on absolute joint angle values.

All metrics were computed for all tasks under the optimal condition and subsequently extended to the suboptimal conditions. Following the threshold proposed by Jo et al. [28], tasks with an nRMSE below 20% in the optimal condition were reported in the main body of the manuscript, whereas the results for the remaining tasks are summarized in the Supplementary Materials. This design choice was based on the assumption that tasks showing insufficient agreement under optimal acquisition conditions would be unlikely to provide reliable measurements under more challenging setups, such as increased camera distance or seated execution. Therefore, the detailed comparison across conditions presented in the main manuscript is restricted to movements that demonstrated acceptable accuracy in the optimal condition, while the complete results for all tasks are provided in the Supplementary Materials.

#### 2.4.3. Performance Indicators

Three Performance Indicators (PIs) generally employed in the domain of interest have been evaluated: the Range of Motion (ROM), the smoothness [36] and the Time to Peak Velocity (TTPV). The ROM quantifies the amplitude of the motion execution, as reported in Equation (4).

(4) $$ROM=\theta_{max}-\theta_{min}$$

The smoothness was computed as the log dimensionless Jerk (LDLJ) according to Equation (5), where T represents the movement duration [38]. The choice of LDLJ was preferred to frequency-domain metrics such as SPARC because SPARC requires uniformly sampled signals. Since the Azure Kinect acquisition frequency is not perfectly constant, the resampling required to obtain uniformly sampled trajectories may alter the spectral content of the signal through interpolation effects, potentially biasing the estimation of frequency-domain smoothness metrics. Although LDLJ is known to be more sensitive to measurement noise, it was considered less affected by this specific limitation.

(5) $$LDLJ=\ln\left(\frac{T^{5}}{ROM^{2}}\int_{0}^{T}{\left(\frac{d^{3}\theta \left(t\right)}{dt^{3}}\right)}^{2}dt\right)$$

The TTPV is an indicator of motion efficiency and was computed as the percentage of the motion execution needed to reach the maximum velocity.

#### 2.4.4. Statistical Analysis

To evaluate the impact of suboptimal tracking conditions on acquisition performance, accuracy metrics were compared using two one-sided *t*-tests (TOSTs) with Benjamini–Hochberg correction. The equivalence bounds for the test were set at ±5 both for the RMSE and nRMSE of angles and velocities and to ±0.1 for correlation. Performance indicators, derived from both the AK and the OS, were computed for each repetition and the median value per subject was used for the subsequent statistical analysis. Specifically, the normalized differences (nDelta) of the performance indicators derived from the OS and AK, respectively, were computed according to Equation (6) and compared across conditions following the same statistical analysis presented for the accuracy metrics. The equivalence bounds for ROM were set at ±5, the one for smoothness at ±0.1 and the one for TTPV at ±10%

(6) $$nDelta=\frac{PI_{OS}-PI_{AK}}{PI_{OS}}\cdot 100$$

All tests were conducted at a significance level of 0.05. Additionally, Bland–Altman analysis was performed for ROM, smoothness, and TTPV. Finally, the Intraclass Correlation Coefficient (ICC) was computed to evaluate the reliability of the performance indicators extracted from either acquisition system. Specifically, ICC(1,6) was computed considering the six repetitions for the sixteen subjects. Following [39], ICC was considered excellent when above 0.9, good when between 0.75 and 0.9, moderate between 0.5 and 0.75, and poor if lower than 0.5. The interclass correlation coefficient, namely ICC(2,1), was also computed to further evaluate agreement between performance indicators and results are reported in the Supplementary Materials.

## 3. Results

### 3.1. Participants

In total, 16 subjects participated in this study, 9 males and 7 females, all with Caucasian ethnicity and with an average age of 25.5±2.9 years. Their mean height was 173±8 cm and their mean mass was 66.2±9.5 kg. All subjects were within normal weight.

### 3.2. Joint Angles

**Figure 5:**
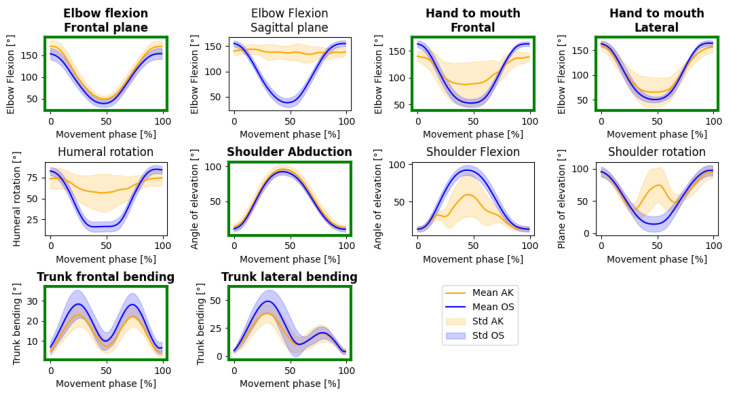
Kinematic traces for all the considered motor tasks in the optimal condition. The blue line represents the data acquired by the optoelectronic system (OS) while the orange line represents the one tracked by Azure Kinect (AK). The mean (and standard deviation) traces across subjects are reported. The traces with the green border and **bold title** refer to the motor tasks with nRMSE <20%.

Elbow, shoulder, and trunk angles were extracted from both tracking systems for each condition. Figure 5 illustrates the mean (and standard deviation) of the kinematic traces in the optimal condition. Notably, gestures performed in the camera plane (e.g., elbow flexion or shoulder abduction) exhibit close alignment between the two traces, whereas movements performed in planes perpendicular to the camera’s one present substantial discrepancies. These errors are particularly pronounced in motions involving intrinsic self-occlusions, such as shoulder rotation, where a distinct error peak emerges at the midpoint of the movement phase, when the wrist, elbow, and shoulder joints are aligned with the AK optical axis. The same happens for the flexion movements in the sagittal plane, where the elbow keypoints is barely recognized. Both frontal and lateral trunk bending angles are effectively measured by the AK.

### 3.3. Joint Angular Velocities

**Figure 6:**
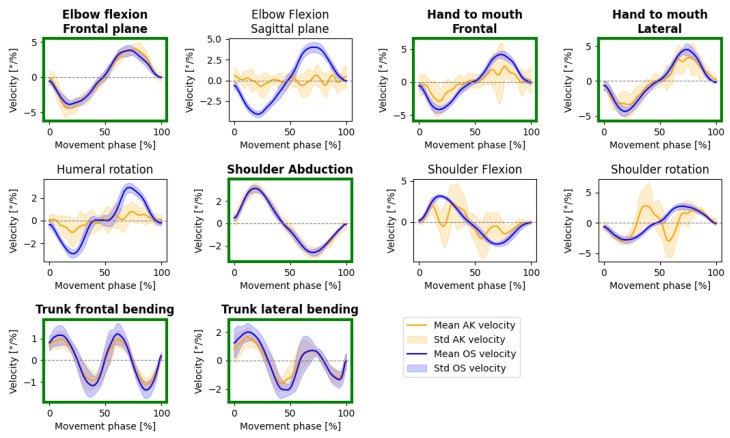
Joint angular velocity profiles for all the considered motor tasks in the optimal condition. The blue line represents the velocity derived from data acquired by the optoelectronic system (OS) while the orange line represents the one from Azure Kinect (AK). The mean (and standard deviation) traces across subjects are reported. The traces with the green border and **bold title** refer to the motor tasks with nRMSE <20%.

Starting from the joint-angle trajectories, joint angular velocity profiles were computed as the first derivative of the time-normalized kinematic traces. Figure 6 reports the mean and standard deviation of the angular velocity profiles for the optimal condition. Consistent with the findings obtained from the joint-angle trajectories, the tasks that showed good agreement between systems also exhibited a high degree of similarity in their velocity profiles. In particular, elbow flexion, hand-to-mouth lateral, shoulder abduction, and trunk bending tasks showed comparable waveform shapes and timing of the main velocity peaks. Minor discrepancies were observed for the hand-to-mouth frontal task. Although the timing of the main velocity peaks was preserved, the Azure-based measurements tended to underestimate the magnitude of both the maximum and minimum velocities. Furthermore, a larger inter-subject variability was observed for this task, as reflected by the wider standard deviation bands. Conversely, tasks that exhibited poor agreement in the joint-angle trajectories also showed substantial discrepancies in the corresponding velocity profiles. This observation suggests that the errors observed in movements performed outside the camera plane cannot be solely attributed to constant angular offsets, since differentiation reduces the influence of constant offsets.

### 3.4. Accuracy Metrics

The RMSE were computed both for joint angles and joint angular velocities across conditions and results are reported in Table 2. Considering joint angles, median RMSE values exceeded 20° only for elbow flexion in the sagittal plane. In contrast, the lateral hand-to-mouth, shoulder abduction, and trunk bending tasks yielded RMSE values below 10°, indicating good agreement between the two measurement systems. Velocity profiles showed trends similar to those observed for joint-angle RMSE, with the tasks exhibiting the lowest relative errors under optimal conditions corresponding to those identified as most accurate in the joint-angle analysis. However, the degradation in performance under suboptimal conditions was more pronounced for velocity-based measures than for joint angles.

Subsequent analyses are therefore reported in the main body of the manuscript only for motor tasks with an nRMSE below 20%, in order to evaluate their performance across different conditions. These tasks included elbow flexion in the frontal plane, both hand-to-mouth tasks, shoulder abduction, and both trunk bending tasks. The results for the remaining tasks are reported in the Supplementary Materials.

**Figure 7:**
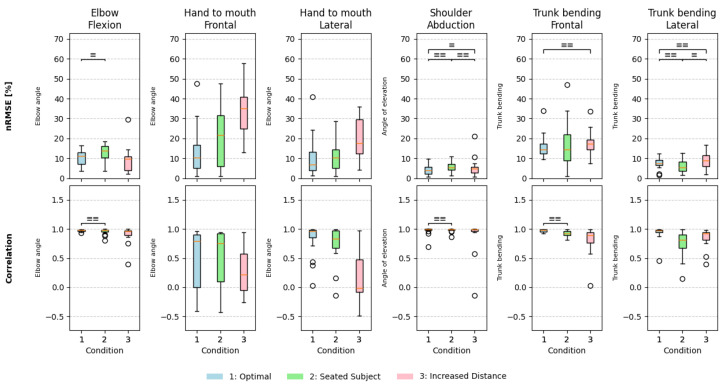
nRMSE and correlation of the joint angles in optimal and suboptimal conditions for tasks with nRMSE < 20% in the optimal condition. TOST test with Benjamini–Hochberg correction was applied. ≡ indicate p<0.05; ≡≡ indicate p<0.01.

Figure 7 complements Table 2, summarizing the nRMSE and Pearson’s correlation coefficients obtained for the motor tasks that met the acceptance criterion (nRMSE < 20% under optimal conditions) across the three experimental conditions. The comparison for the remaining tasks is reported in Figure S1 of the Supplementary Materials. Overall, shoulder abduction and both trunk bending tasks exhibited the lowest errors, with median RMSE values generally below 10° and correlation coefficients close to 1 across all conditions. Elbow flexion showed slightly higher errors, although median RMSE and nRMSE remained below the acceptance threshold in all conditions. The hand-to-mouth tasks displayed the largest variability and the highest error values. In particular, the increased-distance condition resulted in higher RMSE and nRMSE values, with median RMSE exceeding 20° and reaching 40° for the frontal hand-to-mouth task. Correspondingly, correlation coefficients were lower and more dispersed for both hand-to-mouth movements compared with the other tasks.

The equivalence analysis reached significance between conditions for shoulder abduction and both trunk bending tasks (p<0.01 for most comparison but for the increased distance nRMSE with p=0.047). A similar result was observed for elbow flexion when comparing the optimal and seated conditions (p=0.04). In contrast, no equivalence was identified for the hand-to-mouth tasks, which showed greater variability and reduced accuracy, particularly in the increased-distance condition.

For tasks with non-acceptable nRMSE values in the optimal condition, no improvement in performance was observed under the suboptimal acquisition conditions. Furthermore, waveform similarity between the two systems remained limited, as indicated by the moderate correlation coefficients. Together, these results suggest that the Azure Kinect has difficulty accurately capturing movements outside the camera plane regardless of the acquisition condition.

**Figure 8:**
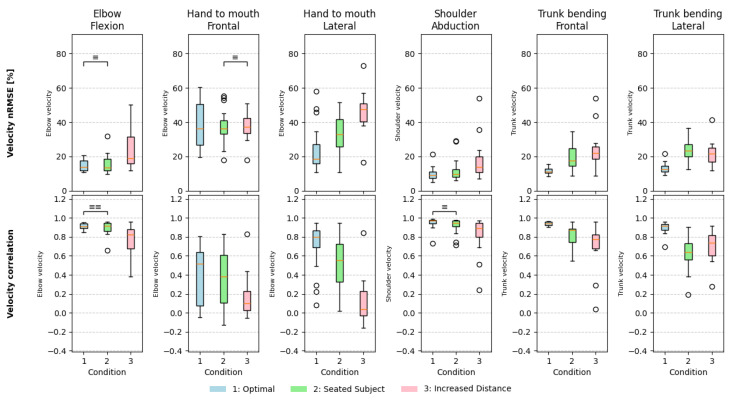
nRMSE and correlation of joint angular velocity in optimal and suboptimal conditions for tasks with nRMSE < 20% in the optimal condition. TOST test with Benjamini–Hochberg correction was applied. ≡ indicate p<0.05; ≡≡ indicate p<0.01.

The same comparison was extended to angular velocity, with results reported in Table 2 and Figure 8 for tasks which satisfied the acceptance criterion and in Figure S2 of the Supplementary Materials for tasks which did not meet such a criterion. Tasks characterized by movements predominantly occurring within the camera plane, such as elbow flexion, shoulder abduction, and trunk bending, exhibited relatively low velocity RMSE values (mostly below 2°/%) and high correlations, with values constantly around 0.9 in the optimal condition. Elbow flexion presents similar results across conditions, especially the seated subject case, for which equivalence testing revealed statistical similarity in all parameters (p=0.01 in nRMSE and p<0.01 for correlation). Hand-to-mouth frontal and hand-to-mouth lateral movements showed substantially larger velocity errors, which may be especially due to the different estimation of maximum and minimum velocities noted from the analysis of the joint angular velocity profiles. Moreover, the hand-to-mouth frontal task exhibited the highest variability across subjects and conditions, as reflected by the large interquartile ranges. Although moderate median correlations were obtained in the optimal and seated subject conditions, performance deteriorated markedly in the increased distance condition, which indicates that while absolute values are consistent, the waveforms deviate with increasing distance. Similarly, the hand-to-mouth lateral task showed a progressive increase in RMSE and nRMSE together with a reduction in correlation in the suboptimal conditions. Shoulder abduction and trunk bending tasks demonstrated the highest robustness to experimental condition changes. Equivalence was found only in the seated subject condition (p=0.01). Concerning trunk bending tasks, the velocity traces showed good agreement between systems as reflected in the low RMSE and nRMSE values and the high correlation coefficients observed across all conditions. Although a progressive increase in error was observed from the optimal condition to the increased distance condition, the overall deterioration remained limited, and the velocity waveform shape was largely preserved. This effect is larger in the lateral trunk bending than the frontal one. Finally, the tasks that did not meet the acceptance criterion exhibited relatively high nRMSE values, typically around 40% across conditions, together with low correlation coefficients. An exception was observed for the shoulder rotation angle when considering joint angular velocity profiles. In this case, nRMSE values were substantially lower (around 10%) and waveform similarity was high, with excellent correlation coefficients in both the optimal and seated-subject conditions. Moreover, equivalence testing demonstrated statistically significant equivalence between these two conditions, suggesting that the temporal evolution of the movement was better preserved than its absolute angular magnitude.

### 3.5. Performance Indicators

ROM, movement smoothness, and Time to Peak Velocity were computed for each motor task. To quantify the error, the normalized differences between the performance indicators obtained from the two measurement systems were calculated.

**Figure 9:**
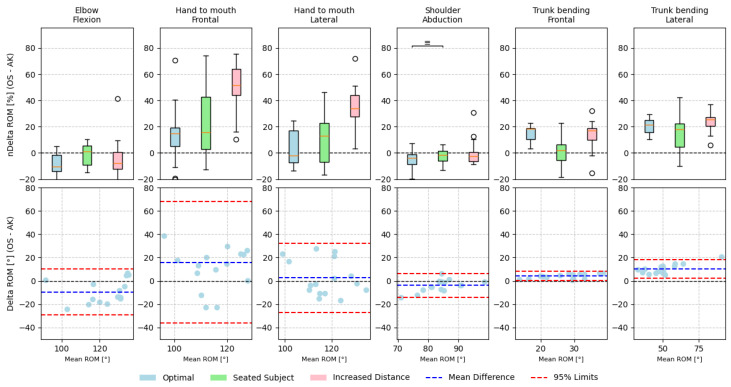
The upper panel reports the range-of-motion normalized error distribution in optimal and suboptimal conditions. TOST test with Benjamini–Hochberg correction was applied. ≡ indicate p<0.05. The lower panel depicts the Bland–Altman plots of the ROM for each motor task in the optimal condition.

Figure 9 presents the normalized difference in ROM measured by the OS and AK systems (nΔROM [%]) across the different experimental conditions for tasks that exhibited an nRMSE below 20% in the optimal condition.

The smallest biases are observed for shoulder abduction, where median normalized errors remain close to zero across all conditions and the Bland–Altman plots show narrow limits of agreement. This finding suggests robust ROM estimation regardless of the environmental setup. This observation is further supported by the statistical equivalence identified between the optimal and seated subject conditions for the shoulder abduction task (p=0.04). For elbow flexion, the median differences also remain relatively small (around 10% in the optimal and increased distance conditions and around 0% in the seated subject condition), although a moderate dispersion of measurements is observed. The hand-to-mouth tasks exhibit the largest variability and the widest limits of agreement in the Bland–Altman analysis, reflecting the greater tracking complexity associated with movements occurring partially in the sagittal plane. In the frontal execution, the increased distance condition shows a marked positive bias, with median normalized errors exceeding 50%, indicating a substantial overestimation of ROM. Similarly, the lateral hand-to-mouth movement shows progressively larger positive errors from the optimal to the increased distance condition, suggesting a strong sensitivity to measurement geometry and sensor placement. For the trunk bending tasks, all conditions show a consistent, although limited, positive bias, indicating a general underestimation of trunk ROM. Notably, the normalized ROM error is markedly reduced in the seated subject condition during frontal trunk bending. This result may be explained by the reduced movement amplitude that occurred when participants performed the task while seated on a stool. The Bland–Altman analysis indicates homoscedasticity across all measured angles, with no evident relationship between error magnitude and ROM magnitude over the observed measurement range.

Concerning the tasks that did not meet the acceptance criterion, the corresponding analysis is reported in Figure S3 of the Supplementary Materials. Overall, these tasks exhibited substantially larger errors, as evidenced by the higher normalized differences and the presence of marked systematic biases in the Bland–Altman analysis. An exception was observed for the shoulder rotation task, which showed a negligible mean bias. However, this apparent agreement should be interpreted with caution, as the corresponding limits of agreement remained very large (approximately 40°), indicating considerable variability between the two measurement systems.

**Figure 10:**
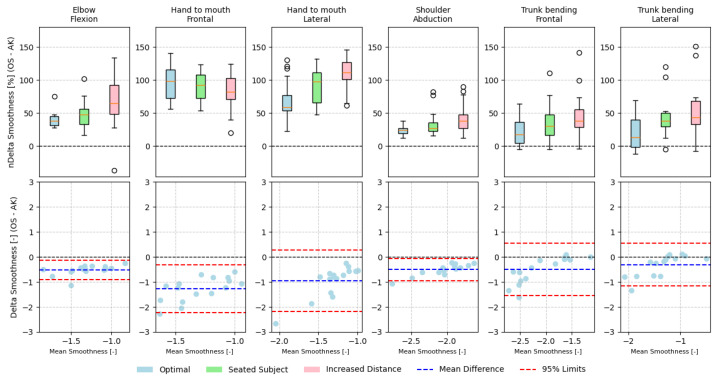
The upper panel reports the smoothness normalized error distribution in optimal and suboptimal conditions. TOST test with Benjamini–Hochberg correction was applied. The lower panel depicts the Bland–Altman plots of the ROM for each motor task in the optimal condition.

The same analytical approach was followed for the smoothness, with results reported in Figure 10 for tasks passing the acceptance criterion. Overall, the AK tends to underestimate movement smoothness, as indicated by the predominantly positive normalized differences observed across all tasks and conditions. The largest normalized differences are observed for the hand-to-mouth tasks, particularly in the frontal and lateral executions, where median percentage errors frequently approach or exceed 100%. Moreover, the increased distance condition generally produces the highest smoothness differences, suggesting that the metric is sensitive to measurement geometry and tracking quality. For shoulder abduction, the normalized differences remain lower than those observed for the hand-to-mouth tasks, although a systematic positive bias is still evident across all conditions. Similarly, elbow flexion shows a consistent underestimation of smoothness, with moderate variability between participants, especially in the optimal condition. The trunk bending tasks also exhibit positive smoothness differences, indicating that the OS system consistently estimates smoother movement profiles than the Azure Kinect. The Bland–Altman analysis reveals a predominantly negative mean difference because smoothness values are expressed on a logarithmic scale and are negative by definition. Across all tasks, the mean differences remain relatively constant over the measurement range, indicating homoscedasticity and the absence of proportional bias. However, wider limits of agreement are observed for the hand-to-mouth tasks, reflecting the larger variability associated with these more complex upper-limb movements. In contrast, elbow flexion, shoulder abduction, and trunk bending tasks exhibit narrower limits of agreement, indicating more consistent agreement between the two systems.

Figure S4 in the Supplementary Materials reports the results for the tasks that did not meet the acceptance criterion. Compared with the tasks included in the main analysis, substantially larger normalized errors were observed, together with wider limits of agreement in the Bland–Altman analysis.

Thus, AK cannot be considered an accurate instrument for assessing upper-limb smoothness based on LDLJ.

**Figure 11:**
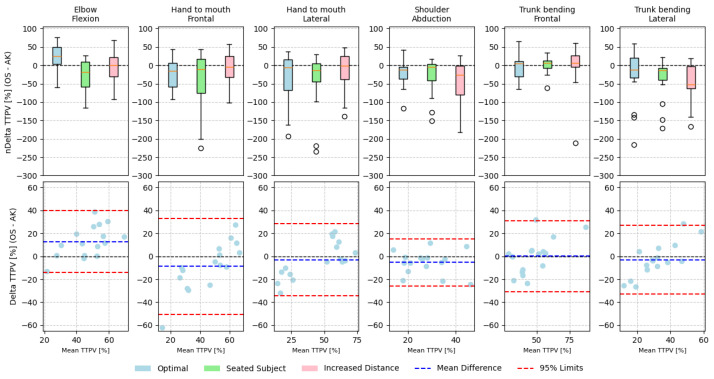
The upper panel reports the TTPV normalized error distribution in optimal and suboptimal conditions. The lower panel depicts the Bland–Altman plots of the ROM for each motor task in the optimal condition.

Concerning the time to peak velocity, Figure 11 presents the normalized differences measured by the OS and AK systems (nDelta TTPV [%]) across different experimental conditions for tasks overcoming the acceptance criterion.

Median normalized differences remain relatively close to zero for most movements, indicating limited systematic bias between the two systems, although an overall overestimation of TTPV timing is observed by negative median difference between the sensing systems across all tasks. Moreover, the TTPV results show great variability, with substantial dispersion observed across participants and tasks, which may suggest that TTPV estimation is sensitive to variations in movement execution and tracking performance. For elbow flexion, the median normalized differences are close to zero across all conditions, although moderate variability is observed. Similar behavior is found for the hand-to-mouth tasks, where the distributions are centered around zero but exhibit considerable dispersion, with several extreme negative outliers, indicating occasional substantial disagreement between the systems. The Bland–Altman analysis shows the widest limits of agreement, reflecting the greater variability associated with these complex upper-limb tasks. Shoulder abduction exhibits a tendency towards negative normalized differences, particularly in the increased distance condition, suggesting a systematic shift between the measurements provided by the two systems. The variability also increases under non-optimal acquisition conditions. For trunk bending tasks, median normalized differences remain close to zero in all conditions, although the presence of some negative outliers is evident in the lateral trunk bending. While no pronounced trend is consistently observed across all movements, AK demonstrates a tendency to overestimate TTPV when the true values are low, while underestimating TTPV when the absolute values increase, especially in trunk bending and hand-to-mouth tasks. This suggests a proportional bias where the magnitude and direction of the difference depends on the actual TTPV value being measured. Overall, the results indicate that TTPV measurements derived from the AK system show acceptable agreement with the OS reference system across most tasks, while it decreases for more complex movements, particularly the hand-to-mouth tasks.

Results related to the tasks that did not meet the acceptance criterion are reported in Figure S5 of the Supplementary Materials. Unlike the other performance indicators, TTPV showed acceptable validity even for these tasks, suggesting that temporal features of the movement may be preserved even when joint-angle trajectories are not accurately reconstructed. Nevertheless, all tasks exhibited a pronounced increase in error with increasing TTPV magnitude, as evidenced by the proportional bias observed in the Bland–Altman analysis. This finding indicates that, although the timing of velocity peaks can be identified with reasonable accuracy, the agreement between systems deteriorates for movements characterized by larger TTPV values.

To further investigate the findings of the previous analysis, ICC(2,1) was computed and results are reported in Table S1 of the Supplementary Materials. Overall, ROM exhibited the highest levels of agreement, with moderate ICC values across most tasks and conditions. The best agreement was observed for the trunk bending tasks, particularly in the frontal plane, where ICC values consistently exceeded 0.75. Shoulder abduction and elbow flexion also showed moderate agreement, although a reduction was observed in the Increased Distance condition. In contrast, smoothness demonstrated poor agreement across all tasks and conditions, with ICC values generally close to zero and occasionally negative. This finding is consistent with the large normalized errors reported in the main analysis and confirms that the Azure Kinect cannot reliably reproduce smoothness estimates obtained from the reference system. TTPV showed variable agreement depending on the task. Moderate agreement was observed for shoulder abduction and hand-to-mouth lateral movements, whereas poor agreement was found for most of the remaining tasks and conditions. Notably, several negative ICC values were observed, indicating that the variability between the two systems exceeded the variability between subjects. These findings suggest that, although TTPV may exhibit limited systematic bias, its agreement with the reference system remains inconsistent. Overall, the ICC(2,1) analysis confirms that ROM showed the best agreement among the investigated performance indicators, whereas smoothness and, to a lesser extent, TTPV should be interpreted with caution due to their limited agreement with the optoelectronic reference system.

Finally, Table 3 reports the intraclass correlation coefficient, i.e., ICC(1,6), for the performance indicators computed from the two motion capture systems. The OS showed good to excellent reliability for both ROM and smoothness across all tasks and conditions. For the Azure Kinect, excellent reliability was observed for all trunk-related ROM measurements, and good reliability in all conditions for the shoulder joint measurements. Elbow flexion ROM was slightly less repeatable, showing moderate reliability for the frontal hand-to-mouth task at increased distance and poor reliability for the elbow flexion task in the seated subject condition. Concerning smoothness, an overall lower reliability with respect to ROM was observed, although ICC values were still mostly in the good-to-excellent range for both motion capture systems. Exceptions were noted for smoothness computed during the shoulder abduction tasks and the frontal hand-to-mouth task where reliability was moderate to poor. With regard to TTPV, both systems demonstrated very limited reliability. The poor consistency observed even with the OS system indicates that this indicator is inherently variable across repetitions, including when assessed with the gold-standard method. Such variability is most likely attributable to the absence of controlled movement velocity, which resulted in natural fluctuations in execution speed across trials.

## 4. Discussion

Azure Kinect is increasingly used for motion analysis in rehabilitation settings, yet its accuracy for upper-limb assessment under clinically relevant conditions remains insufficiently investigated. While previous studies evaluated upper-limb kinematics under controlled experimental setups [6,33,34], this study examined the effects of seated execution and increased camera-to-subject distance, conditions frequently encountered in clinical practice. Furthermore, beyond joint-angle accuracy, we assessed clinically relevant metrics including ROM, smoothness, TTPV, and their repeatability, providing a more comprehensive evaluation of the practical applicability of Azure Kinect for rehabilitation assessment in ecological environments. Our findings indicate that AK accurately captures movements occurring in the frontal plane—corresponding to the camera view—showing strong agreement with the gold-standard optoelectronic system. However, its performance declines for motions in the sagittal and transverse planes (i.e., outside the camera plane), where it struggles to reconstruct the full kinematic profile. Notably, humeral rotation, despite occurring in the camera plane, is poorly measured, with the system detecting minimal to no motion. This may be due to occlusion of key anatomical landmarks, such as the shoulder and clavicle ones, which are critical for the estimation of the joint angle of interest. These results align with previous findings on the upper limb conducted with the previous Kinect V2 as Cai et al. [29] reported angular errors of approximately 6° for shoulder abduction/adduction and approximately 10° for movements performed perpendicular to the camera plane, while Faity et al. [32] reported relative errors around 20% during seated reaching tasks. In the present study, shoulder abduction achieved a median RMSE of 3.4°, whereas movements involving substantial out-of-plane components reached nRMSE values above 30%, confirming that movement direction relative to the camera remains a major determinant of tracking accuracy [30]. Similar results were also found with Azure Kinect. Ivorra et al. [34] reported accurate shoulder abduction reconstruction but substantial errors for elbow flexion and shoulder rotation as well as by Brambilla et al. [6], who identified shoulder and elbow flexion as the most challenging movements. Similarly, in the present study, shoulder abduction achieved a median RMSE of 3.4° and nRMSE of 4.0%, whereas elbow flexion performed outside the camera plane reached a median RMSE of 39.7° and nRMSE of 31.1%. Concerning the lower limb, authors report that the best tracking accuracy was achieved when movements occur in the camera plane, which aligns with our results. However, they also noted that movements occurring on other planes were effectively captured, which may be attributed to the training data used in the Azure Kinect body tracking system, likely enriched with numerous observations of walking subjects and thus optimized for gait-related movements [24].

To quantitatively assess validity, RMSE and nRMSE were employed. Agreement was considered acceptable according to the criterion proposed in [28]. These tasks were therefore selected for detailed discussion in the main manuscript, while the corresponding results for the remaining movements are reported in the Supplementary Materials. The comparison across acquisition conditions focused on determining whether the accuracy observed under optimal conditions was maintained when tracking was performed under more challenging scenarios, namely with the participant seated on a stool and positioned at a greater distance from the camera than recommended.

Overall, RMSE, nRMSE, and Pearson’s correlation coefficients exhibited similar behavior across conditions. Furthermore, statistical equivalence between conditions was observed for the shoulder abduction and trunk bending tasks, as well as for elbow flexion in the seated condition. These findings suggest that the accuracy achieved under optimal conditions is largely preserved even when tracking is performed under less favorable circumstances. In contrast, the hand-to-mouth tasks showed the largest decrease in accuracy, with median percentage errors reaching up to 40% in the increased distance condition. This movement is performed outside the primary anatomical planes, which may contribute to reduced tracking accuracy. Additionally, at greater distances, even small changes in the participant’s orientation can increase the likelihood of partial occlusions. In particular, the wrist may partially obscure the shoulder during movement execution, leading to less reliable keypoint detection and larger estimation errors, even under otherwise nominal conditions.

To further investigate the performance of the Azure Kinect, the analysis was extended to joint angular velocity profiles obtained as the first derivative of the time-normalized joint-angle trajectories. Because differentiation reduces the influence of constant angular offsets, velocity-based metrics provide a complementary assessment of system performance by emphasizing the dynamic characteristics of movement rather than absolute joint positions. Overall, the velocity analysis yielded results consistent with those obtained from the joint-angle trajectories. Tasks characterized by movements predominantly occurring within the camera plane, such as elbow flexion, shoulder abduction, and trunk bending, exhibited low velocity RMSE values, relatively low nRMSE values, and high correlation coefficients across all conditions, indicating good agreement between the markerless and reference systems. Conversely, larger discrepancies were observed for the hand-to-mouth tasks and for movements involving substantial out-of-plane components. These tasks showed increased RMSE and nRMSE values together with lower correlation coefficients, particularly in the increased distance condition. Such errors are mainly related to the magnitude of the estimated velocity, while the timing of velocity peaks is maintained.

**Figure 12:**
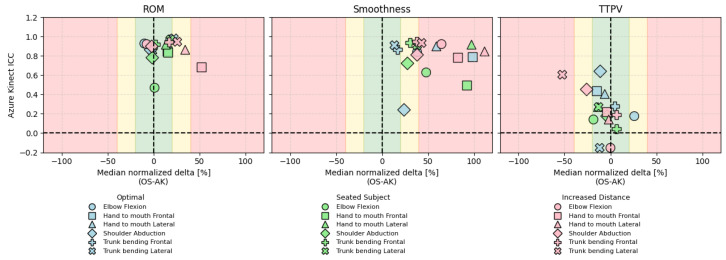
Summary of validity and reliability of the performance indicators derived from the Azure Kinect across all motor tasks and experimental conditions. The x-axis reports the mean normalized difference between the Azure Kinect and the optoelectronic system, expressed as a percentage of the reference value, and provides an indication of validity. The y-axis reports the intraclass correlation coefficient (ICC(1,6)) computed from the Azure Kinect measurements and quantifies reliability. Each marker represents a specific motor task and acquisition condition. Green areas represent good accuracy with absolute median normalized deltas <20%, yellow areas represent medium accuracy with absolute median normalized deltas between 20% and 40%, while red areas represent bad accuracy levels with absolute median normalized deltas > 40%.

Three performance indicators commonly employed in clinical motion analysis—Range of Motion (ROM), smoothness, and Time to Peak Velocity (TTPV)—were further evaluated in terms of validity and reliability. Figure 12 provides a summary of these results by jointly reporting the mean normalized difference between the Azure Kinect and the reference system (validity) and the corresponding ICC values (reliability) across all tasks and experimental conditions. Indicators located closer to the origin of the x-axis and with higher ICC values can be considered more accurate and repeatable. The inaccuracies identified in the kinematic comparisons extend to ROM: the average error of this performance indicator for the frontal hand-to-mouth task in the optimal condition is slightly below 20% of the actual ROM value, and significantly increases to 50% at greater distances. Furthermore, the repeatability of this task is poor in the increased distance case, emphasizing the need to control for camera distance when assessing it. A similar trend is observed in the lateral hand-to-mouth task, though the error is negligible in the optimal condition and the reliability remains good even in the suboptimal ones. Indeed, although occlusions also occur in the lateral execution, the initial and final postures, which are critical for ROM computation, are reliably captured. These observations align with existing literature emphasizing that an increased distance from the camera has a negative impact on the tracking accuracy [23]. Furthermore, no pronounced differences were found between the optimal and seated subject conditions, suggesting that ROM assessment can be performed while seated at a table without substantially compromising measurement accuracy. This observation is particularly supported by the statistical equivalence identified for the shoulder abduction task and by the comparable Bland–Altman agreement observed across conditions. Interestingly, for frontal trunk bending, the seated condition resulted in smaller normalized ROM errors than the optimal condition. This finding may be explained by the reduced movement amplitude associated with performing the task while seated, which likely facilitates tracking by the AK system. Similar observations have been reported by Antico et al. [1]. Results from the smoothness metric highlight the Azure Kinect’s limitations in assessing jerk-related aspects, as the error ranges up to 100% of the actual smoothness value. In fact, although most tasks show ICC(1,6) values above 0.75, errors are consistently non-negligible. This issue is likely due to the amplification of measurement noise during numerical differentiation. Since jerk is the third derivative of position, even minor inaccuracies in joint tracking—arising from sensor noise, temporal resolution limits, or occlusions—are significantly magnified, compromising the reliability of jerk estimates obtained from AK data. The same limitation was found also in AI-powered systems based on multiple RGB cameras [40]. Furthermore, the estimated smoothness may have been influenced by the low-pass filtering applied during signal processing, as jerk-based metrics are particularly sensitive to filtering choices. Future studies should therefore include sensitivity analyses to evaluate the impact of preprocessing parameters, such as cutoff frequency selection, on smoothness quantification and potentially include other smoothness parameters, such as SPARC. Finally, TTPV measures showed good agreement between the two systems, indicating the substantial accuracy of AK even under suboptimal conditions. This reinforces the findings related to the velocity analysis as errors are related to the identification of the amplitude of velocity peaks and not to timing aspects. However, the estimated parameter exhibited high variability and very limited reliability, which limits the strength of this finding. Notably, the poor repeatability observed even with the gold-standard OS suggests that this limitation may have arisen from the inherent variability of the gestures themselves rather than from measurement systems. Additionally, subjects were not instructed to perform velocity-controlled tasks, which may have contributed to natural variations in execution speed across repetitions.

### 4.1. Suggestions for the Clinical Practice

Based on the findings of this validation study, some preliminary consideration for clinical application can be proposed. The Azure Kinect, combined with its proprietary SDK, showed promising performance in the assessment of upper-limb Range of Motion during simple motor tasks. To ensure accurate tracking, particular attention should be paid to camera positioning, with movements ideally occurring within the plane of the sensor’s field of view. Importantly, the performance of the AK system remained largely consistent in the seated condition, supporting its potential applicability in populations unable to perform assessments while standing, such as wheelchair users, as well as during the administration of clinical scales that are commonly performed while seated at a table. Similarly, increasing the camera-to-subject distance had a limited impact on most of the evaluated kinematic parameters, indicating a reasonable robustness of the system to changes in the acquisition setup. However, tasks involving partial limb occlusions or substantial out-of-plane motion showed reduced accuracy and repeatability under these conditions. Furthermore, the velocity-based analysis revealed a more pronounced degradation in performance at larger acquisition distances, with increased RMSE and nRMSE values and reduced correlations for several tasks. This suggests that, although joint-angle estimation remains relatively robust, as also proved in the previous Kinect v2 validations [30], the accurate reconstruction of movement dynamics is more sensitive to acquisition distance. Consequently, camera placement should be carefully considered when the assessment focuses on velocity-related parameters or movement quality rather than solely on joint angle measurements. These observations may support the use of the sensor in more ecological rehabilitation scenarios, including tasks in which patients move freely within the environment while performing concurrent activities [25]. However, these hypotheses should be validated in clinical populations, where movement patterns, compensatory strategies, and pathological postures may further influence tracking performance. Finally, even under optimal experimental conditions and for movements occurring on the camera planes, errors reach 10–15°, which may compromise the ability of the system to detect the relatively small changes typically observed in pre- and post-treatment evaluations. Therefore, caution should be exercised when interpreting small longitudinal variations in clinical settings. These results align with previous findings on Kinect v2, concluding that the applications previously adopting that sensors can leverage Azure Kinect with expected similar performances.

### 4.2. Limitations

Despite the consistent findings, this work has several limitations. First, the study was conducted exclusively at self-paced movement speeds, while faster or slower motions could also be relevant, as execution speed may influence measurement accuracy. This extension would be particularly beneficial for evaluating the reliability of speed-related parameters such as TTPV. Second, the participant cohort consisted exclusively of healthy individuals. Although this allowed a controlled evaluation of the Azure Kinect under different acquisition conditions, the results may not directly generalize to patients with neurological or musculoskeletal impairments. Future studies should therefore include clinical populations to better assess the applicability of the system in rehabilitation contexts. Moreover, the experimental protocol focused on predefined upper-limb movements and did not include reaching or object-interaction tasks, which are frequently used in rehabilitation assessment. Although these tasks can be decomposed into the motor primitives presented in this study, some further self-occlusions may be present when subjects perform activities of daily living, potentially worsening the acquisition accuracy. In addition, the analysis was limited to joint-angle kinematics and clinically derived metrics, without considering endpoint kinematics. Finally, previous studies have reported potential cross-talk effects between the Azure Kinect and optoelectronic systems during concurrent acquisitions, which may affect the validity of the comparison [23,41]. These effects were not explicitly addressed in the present study and may therefore have contributed to an overestimation of the observed errors. In addition, possible infrared interference between the two systems cannot be excluded. An attempt to mitigate these issues consisted in reducing the number of markers used for validation. However, this approach may in turn have introduced systematic offsets, since the selected markers did not perfectly coincide with the anatomical keypoints estimated by the Azure Kinect. Future studies could address these limitations by employing inertial measurement units (IMUs) as reference systems, thereby avoiding the optical interference that may arise during concurrent acquisitions with optoelectronic cameras and depth sensors. In addition, IMUs can be directly attached to body segments and do not rely on line-of-sight constraints, potentially enabling more robust validation. Their portability and ease of integration may also facilitate validation protocols outside laboratory settings, allowing the evaluation of markerless systems during more realistic rehabilitation scenarios and daily-life activities. Additionally, the integration of augmented reality tools may help constrain subjects’ movements and improve task repeatability [42]. Finally, the Azure Kinect and optoelectronic system were not hardware-synchronized, and kinematic traces were aligned during post-processing. Although this procedure enabled direct comparison between systems, synchronization errors may have affected the estimation of time-dependent parameters.

Future studies should also include a systematic comparison between the Azure Kinect and other AI-based skeleton extraction systems relying only on RGB cameras (e.g., OpenPose or MediaPipe). Such benchmarking will be essential to validate the Azure Kinect against recent low-cost solutions and easy-to-use processing pipelines. This would be especially relevant given the discontinuation of Azure Kinect. Nevertheless, the Azure Kinect sensor architecture and body-tracking SDK have been transferred to the Orbbec Femto camera platform, suggesting that many of the findings reported in the present study may remain applicable to current RGB-D systems relying on the same tracking framework. Moreover, the identified challenges related to occlusions, movement plane, and camera positioning are not specific to Azure Kinect and may inform the validation of newer markerless motion-capture technologies, including RGB-based approaches such as OpenPose, and MediaPipe.

## 5. Conclusions

This validation study demonstrated that, under specific constraints, the Azure Kinect combined with its proprietary SDK is a promising low-cost solution for quantitative motor assessment of selected upper-limb kinematic outcomes under specific acquisition conditions, including some clinically relevant suboptimal scenarios. The system showed the best overall performance for relatively planar movements, particularly when the motion occurred within the camera plane, supporting its use for the assessment of range of motion and other kinematic outcomes during standardized rehabilitation exercises. Conversely, caution is warranted when evaluating complex multi-plane movements or deriving highly dynamic temporal metrics, such as TTPV and smoothness, for which larger errors and lower repeatability were observed. These findings suggest that the Azure Kinect is a promising low-cost solution for quantitative motor assessment in ecological clinical environments, provided that task selection, camera positioning, and the interpretation of temporal performance metrics are carefully considered.

## Figures and Tables

**Table 1:** For each task, the angle of interest, defined according the ISB convention, is reported. The vectors used for angle computation are reported in the last column.

Task	Angle of Interest	Vectors
Elbow flexion frontal plane	Elbow flexion angle	Forearm—Arm
Elbow flexion sagittal plane	Elbow flexion angle	Forearm—Arm
Hand-to-mouth frontal	Elbow flexion angle	Forearm—Arm
Hand-to-mouth lateral	Elbow flexion angle	Forearm—Arm
Humeral rotation	Humeral rotation angle	Forearm—Transverse axis
Shoulder abduction	Angle of elevation	Arm—trunk
Shoulder flexion	Angle of elevation	Arm—trunk
Shoulder rotation	Plane of elevation	Arm—Transverse axis
Trunk frontal bending	Trunk flexion–extension angle	Trunk—Vertical axis
Trunk lateral bending	Trunk lateral rotation angle	Trunk—Vertical axis

**Table 2:** Across-subject median [IQR] of the Root Mean Square Errors (RMSE) of the joint angles and joint angular velocities analysed across condition. Motor tasks with nRMSE <20% are highlighted in bold.

	RMSE on Joint Angle [°]			RMSE on Joint Angular Velocity [°/%]		
Task	Optimal	Seated Subject	Increased Distance	Optimal	Seated Subject	Increased Distance
Elbow flexion (frontal plane)	**14.75 [6.17]**	**16.21 [6.15]**	**12.38 [9.25]**	**1.27 [0.39]**	**1.21 [0.62]**	**1.95 [1.18]**
Elbow flexion (sagittal plane)	39.67 [12.47]	41.75 [16.21]	50.65 [10.77]	3.61 [0.66]	3.40 [0.28]	3.50 [0.28]
hand-to-mouth frontal	**12.36 [12.31]**	23.03 [27.89]	40.82 [16.50]	3.26 [1.60]	3.09 [0.76]	3.25 [0.51]
hand-to-mouth lateral	**7.84 [10.39]**	**12.32 [10.03]**	**20.53 [20.09]**	**1.75 [1.24]**	2.61 [1.39]	4.30 [1.19]
Humeral Rotation	17.35 [22.44]	23.97 [15.78]	36.23 [10.29]	3.26 [1.60]	3.09 [0.76]	3.25 [0.51]
Shoulder abduction	**3.36 [2.94]**	**4.48 [2.24]**	**4.06 [2.76]**	**0.55 [0.20]**	**0.57 [0.30]**	**0.77 [0.69]**
Shoulder flexion	19.13 [15.95]	24.47 [9.00]	31.87 [10.07]	2.29 [0.39]	2.06 [0.29]	2.20 [0.27]
Shoulder rotation	15.44 [11.41]	24.96 [7.82]	29.65 [17.66]	**0.55 [0.20]**	**0.57 [0.30]**	0.77 [0.69]
Trunk bending frontal	**4.11 [1.98]**	**3.21 [2.22]**	**4.81 [1.52]**	**0.37 [0.05]**	**0.43 [0.30]**	0.76 [0.29]
Trunk bending lateral	**3.52 [0.95]**	**1.89 [1.39]**	**4.45 [2.83]**	**0.66 [0.24]**	0.97 [0.33]	1.05 [0.44]

**Table 3:** Intra-class Correlation Coefficients (ICC) for the Range of Motion (ROM), the movement smoothness and the Time to Peak Velocity (TTPV) for the two motion capture systems under investigation (OS: Optoelectronic system, AK: Azure Kinect.) Values are color-coded: excellent reliability (>0.90), good reliability (>0.75), moderate reliability (>0.50), poor reliability (<0.50).

Task	Joint	Condition	ROM		SMOOTH- NESS		TTPV	
			ICC OS	ICC AK	ICC OS	ICC AK	ICC OS	ICC AK
Elbow Flexion	Elbow	Optimal	0.91	0.93	0.91	0.84	0.36	0.18
		Seated Subject	0.97	0.48	0.96	0.63	0.43	0.14
		Increased Distance	0.95	0.93	0.87	0.92	0.53	0.00
Hand-to-mouth Frontal	Elbow	Optimal	0.92	0.85	0.78	0.79	0.61	0.44
		Seated Subject	0.88	0.84	0.86	0.50	0.72	0.00
		Increased Distance	0.85	0.69	0.79	0.78	0.64	0.22
Hand-to-mouth Lateral	Elbow	Optimal	0.95	0.87	0.82	0.90	0.84	0.41
		Seated Subject	0.98	0.92	0.96	0.92	0.75	0.27
		Increased Distance	0.92	0.86	0.90	0.85	0.71	0.15
Shoulder Abduction	Shoulder	Optimal	0.96	0.87	0.95	0.24	0.25	0.64
		Seated Subject	0.97	0.79	0.97	0.72	0.22	0.18
		Increased Distance	0.97	0.90	0.97	0.81	0.12	0.45
Trunk bending Frontal	Trunk	Optimal	0.97	0.97	0.96	0.87	0.49	0.28
		Seated Subject	0.97	0.92	0.96	0.94	0.00	0.05
		Increased Distance	0.97	0.95	0.93	0.95	0.65	0.19
Trunk bending Lateral	Trunk	Optimal	0.98	0.98	0.95	0.91	0.51	0.00
		Seated Subject	0.97	0.97	0.93	0.91	0.47	0.27
		Increased Distance	0.98	0.95	0.96	0.94	0.48	0.61

## Data Availability

The raw data supporting the conclusions of this article will be made available by the authors, without undue reservation.

## Supplementary Materials

The following supporting information can be downloaded at: https://www.mdpi.com/article/10.3390/s26134098/s1, Figure S1: nRMSE and correlation of the joint angles in optimal and suboptimal conditions for tasks with nRMSE > 20% in the optimal condition; Figure S2: nRMSE and correlation of the joint angular velocities in optimal and suboptimal conditions for tasks with nRMSE > 20% in the optimal condition; Figure S3: Range of Motion normalized error and Bland–Altman plots for tasks which did not pass the acceptance criterion. Figure S4: Smoothness normalized error and Bland–Altman plots for tasks with nRMSE > 20% in the optimal condition; Figure S5: Time to Peak Velocity normalized error and Bland–Altman plots for tasks with nRMSE > 20% in the optimal condition; Table S1: Interclass correlation coefficient (ICC(2,1)) between the performance indicators estimated through the Azure Kinect and the optoelectronic system.
