# Supplementary material for: Validation of Azure Kinect for Upper Limb Motion Analysis Under Optimal and Suboptimal Conditions
Source: Sensors (Basel). 2026 Jun 27;26(13):4098. doi: 10.3390/s26134098 (PMC13363823; doi:10.3390/s26134098)
Supplement: Supplementary file 1 [file sensors-26-04098-s001.zip › sensors-4336430-supplementary.pdf]

Supplementary Material**: Validation of Azure Kinect for Upper Limb Motion Analysis Under Optimal and Suboptimal Conditions**

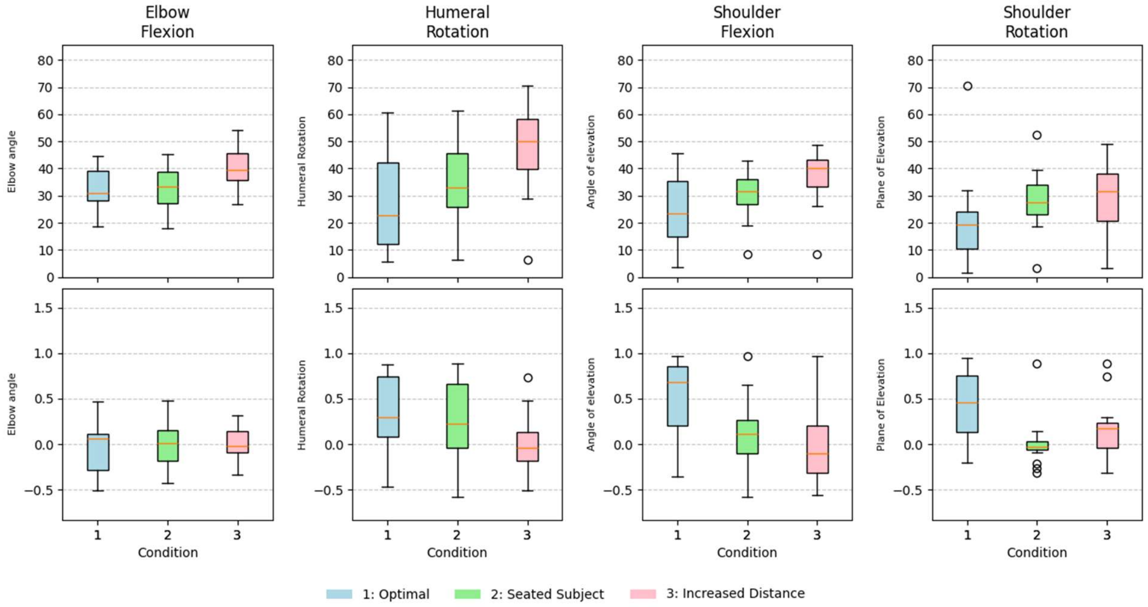

Figure S1: nRMSE and correlation of the joint angles in optimal and suboptimal conditions for tasks with nRMSE>20% in the optimal condition .

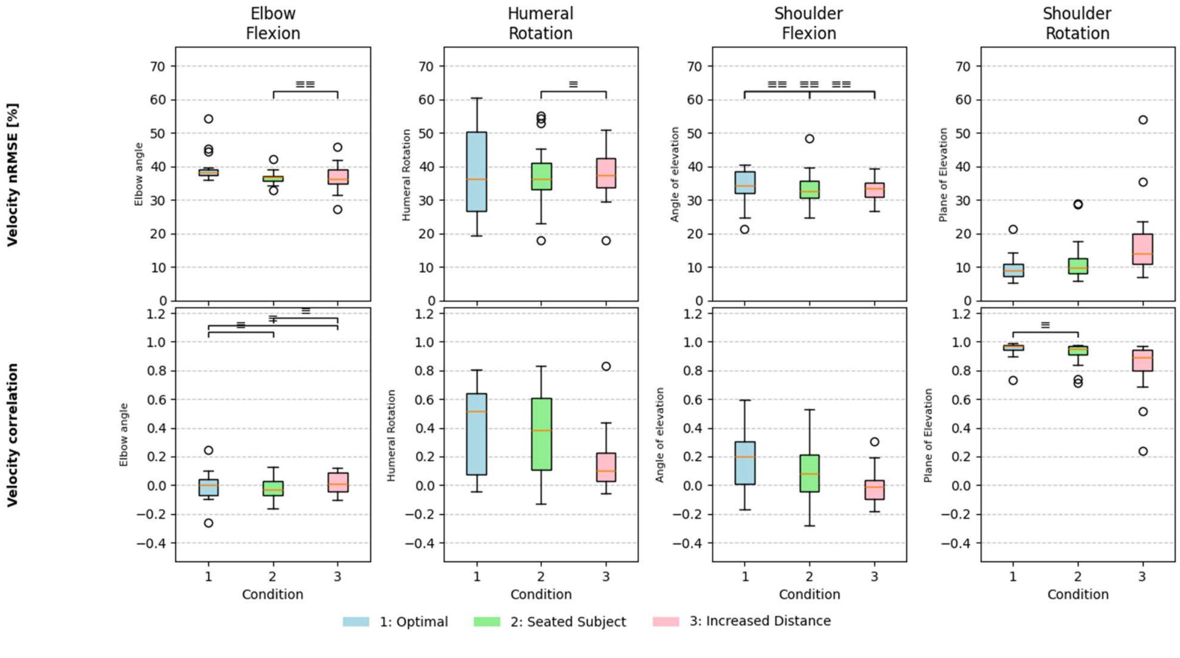

Figure S2: nRMSE and correlation of the joint angular velocities in optimal and suboptimal conditions for tasks with nRMSE>20% in the optimal condition . TOST test with Benjamini-Hochberg correction was applied. ≡ indicate p<0.05; ≡≡

indicate p<0.01.

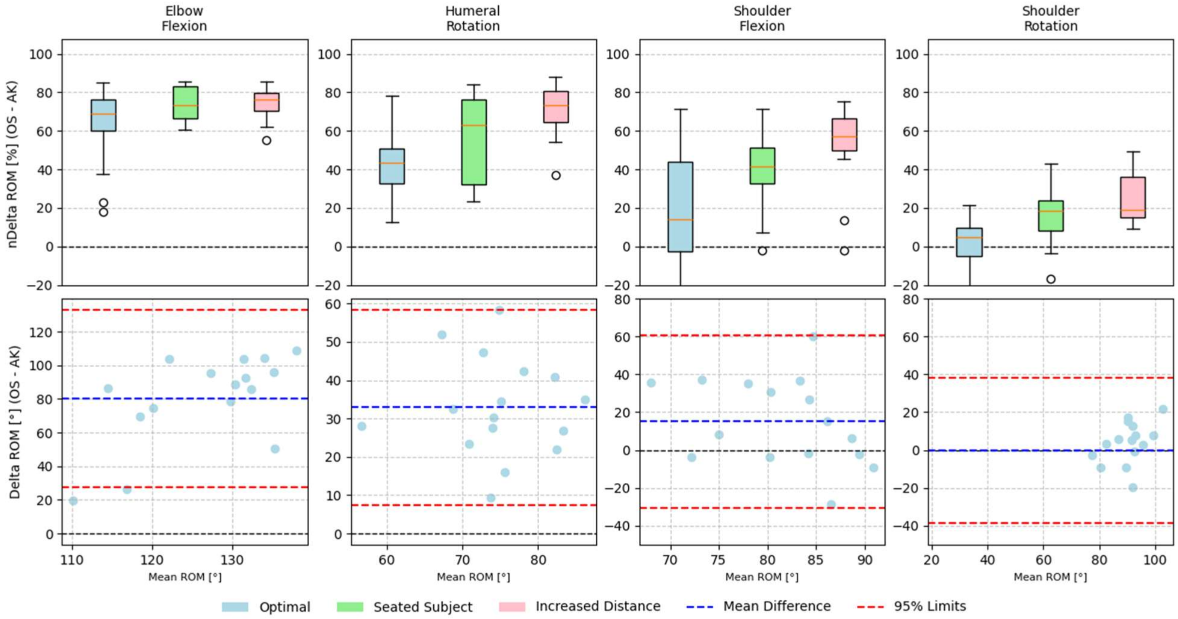

Figure S3: The upper panel reports the Range of Motion normalized error distribution in optimal and suboptimal conditions for tasks which did not pass the acceptance criterion. The lower panel depicts the Bland-Altman plots of the ROM for each motor task in the optimal condition.

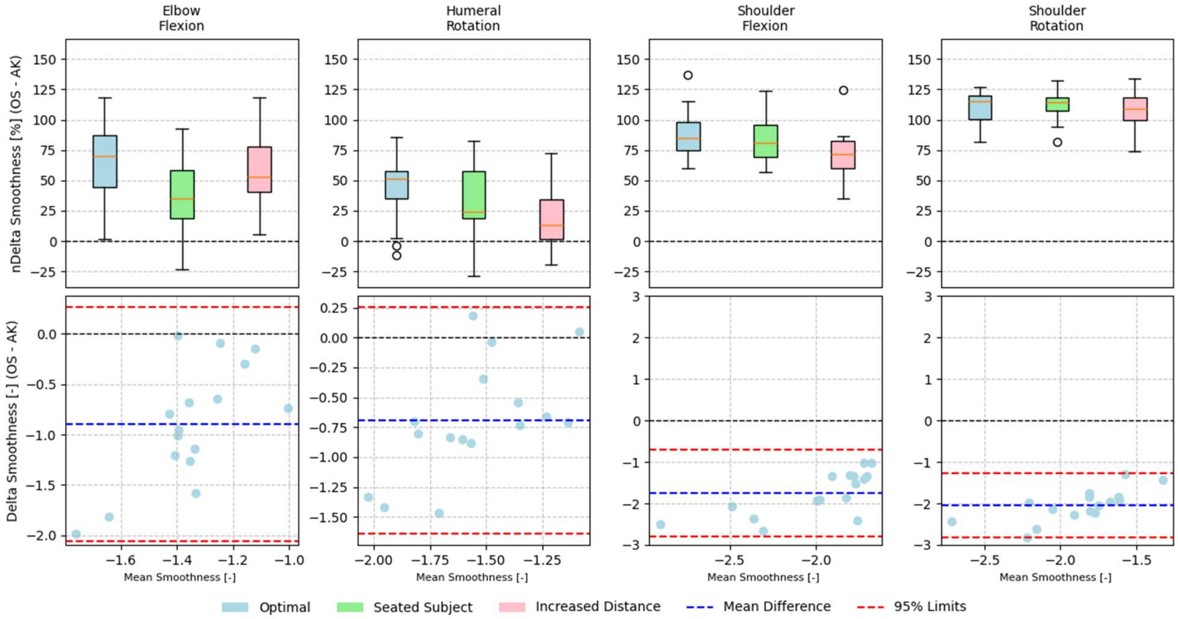

Figure S4: The upper panel reports the Smoothness normalized error distribution in optimal and suboptimal conditions for tasks which did not pass the acceptance criterion. The lower panel depicts the Bland-Altman plots of the Smoothness for each motor task in the optimal condition.

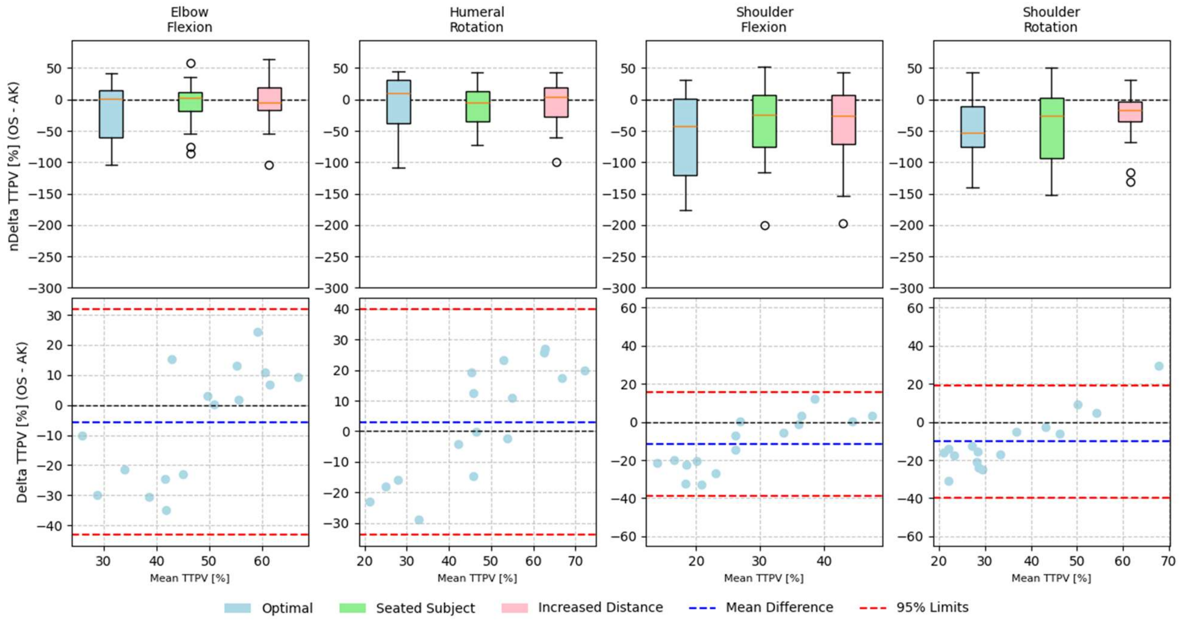

Figure S5: The upper panel reports the Time To Peak Velocity normalized error distribution in optimal and suboptimal conditions for tasks which did not pass the acceptance criterion. The lower panel depicts the Bland-Altman plots of the TTPV for each motor task in the optimal condition.

Task	Joint	Condition	ROM	SMOOTHNESS	TTPV
Elbow Flexion	Elbow	Optimal	0.57	0.22	0.26
		Seated Subject	0.40	-0.14	0.27
		Increased Distance	-0.11	-0.13	-0.11
Hand to mouth Frontal	Elbow	Optimal	0.08	-0.02	-0.08
		Seated Subject	0.08	-0.01	-0.20
		Increased Distance	0.05	-0.06	-0.08
Hand to mouth Lateral	Elbow	Optimal	0.55	-0.13	0.60
		Seated Subject	0.31	-0.04	0.41
		Increased Distance	0.06	-0.02	0.41
Shoulder Abduction	Shoulder	Optimal	0.51	0.15	0.60
		Seated Subject	0.66	0.03	0.38
		Increased Distance	0.46	-0.06	0.17
Trunk bending Frontal	Trunk	Optimal	0.80	0.08	0.37
		Seated Subject	0.88	0.01	0.68
		Increased Distance	0.76	-0.13	0.20
Trunk bending Lateral	Trunk	Optimal	0.69	0.16	0.03
		Seated Subject	0.60	0.11	0.13
		Increased Distance	0.51	0.06	0.47

Table S1: Interclass Correlation Coefficient (ICC(2,1)) between the Performance Indicators estimated through the Azure Kinect and the Optoelectronic System.
